# Overlooked Photochemical Risk of Antimicrobial Fragrances: Formation of Potent Allergens and Their Mechanistic Pathways

**DOI:** 10.3390/toxics13050386

**Published:** 2025-05-10

**Authors:** Xiaolin Niu, Junji Wu, Yi Chen, Na Luo, Yanpeng Gao

**Affiliations:** 1 Guangdong-Hong Kong-Macao Joint Laboratory for Contaminants Exposure and Health, Guangdong Key Laboratory of Environmental Catalysis and Health Risk Control, Institute of Environmental Health and Pollution Control, Guangdong University of Technology, Guangzhou 510006, China; nnxxllin@163.com (X.N.); junjiwu@163.com (J.W.); cy13011501@163.com (Y.C.); ronaluo_1992@163.com (N.L.); 2 Guangzhou Key Laboratory Environmental Catalysis and Pollution Control, Guangdong Basic Research Center of Excellence for Ecological Security and Green Development, School of Environmental Science and Engineering, Guangdong University of Technology, Guangzhou 510006, China; 3 College of Construction and Ecology, Shantou Polytechnic, Shantou 515078, China

**Keywords:** fragrance ingredient, cinnamaldehyde, laser flash photolysis, photodegradation, skin sensitization

## Abstract

Antimicrobial fragrances, commonly found in household and personal care products, are frequently detected in water bodies, yet their environmental fate and transformation mechanisms remain inadequately explored. This study investigates the photochemical transformation of cinnamaldehyde (CA), a representative antimicrobial fragrance, and its consequence for toxicological effects. The results showed that under UV irradiation, 94.6% CA was eliminated within 60 min, with a degradation rate of 0.059 min^−1^. Laser flash photolysis, quenching experiments, and electron paramagnetic resonance spectra identified O_2_^•−^ and ^3^CA^*^ as the important species, contributing 29.4% and 33.6%, respectively, to the transformation process. Additionally, singlet oxygen (^1^O_2_), hydroxyl radicals (^•^OH), and solvated electrons (e_aq_^−^) were involved in mediating the oxidation reactions. These species facilitated photoionization and oxidation, resulting in the formation of five major transformation products, including cis-cinnamyl aldehyde, cinnamic acid, styrene, 1aH-indeno [1,2-b]oxirene), and 1-Oxo-1H-indene. Most of these products were persistent, and exhibited considerable ecotoxicological risks. Specifically, the cinnamic acid and 1-Oxo-1H-indene caused severe skin irritation, while cinnamic acid induced significant eye irritation. Notably, the transformation products demonstrated sensitizing effects on human skin. This study underscores the overlooked ecotoxicological risks associated with the photochemical transformation of antimicrobial fragrances, revealing their potential to generate potent allergens and other harmful byproducts.

## 1. Introduction

Fragrances materials (FMs) are widely used in various foods and personal care products (PCPs), medical products, and industrial chemicals for providing a pleasant scent, such as shampoos, e-cigarettes, and drugs [1,2,3]. Besides providing pleasant scent, several FMs, e.g., cinnamaldehyde (CA), can have exciting antimicrobial properties, and are added to chewing gums, tooth powder, and food packaging materials [4,5]. In addition, CA is commonly used as a corrosion inhibitor in hydraulic fracturing operations [6]. Due to their wide application and society’s overwhelming consumerism, thousands of FMs are being used [7]. For instance, the worldwide usage of individual CA is reported as 100~1000 metric tons annually [8]. Despite FMs having been detected in surface and subsurface water [9], a quantitative evidence synthesis from the environmental PPCP literature indicates that a distinct knowledge gap still exists in FMs [2].

Numerous reports show that antimicrobial FMs may elicit various adverse health effects, such as allergic and irritant dermatitis [10], as well as respiratory irritations (e.g., asthma and rhinitis) [11]. Exposure to antimicrobial FMs, e.g., CA, induces genetic alterations in hepatocytes [12,13,14], and potential toxicity of the product in human embryonic cells and lung cells [15]. A recent review reported that the unprecedented prevalence of depression, cancer, and obesity is linked to the limitless usage of antimicrobial FMs [7]. The acute toxicity of FMs to aquatic organisms ranges from ppb to ppm [16]. Although the concentration of FMs in the aquatic environment is at the ng/L level [17], their potential health risks still cannot be ignored. Even several FMs with low concentrations could impose anti-estrogenic effects, as well as inhibit multixenobiotic resistance in mussels and larval development in marine copepods [18]. Skin sensitization was found in the local lymph node and percutaneous absorption assay in mice and humans, respectively [19,20]. Notably, FMs containing hydrophobic groups often exhibit lipophilic behavior, which is closely related to bioaccumulation potential [21,22]. Particularly, CA was listed as one of the 26 allergenic FMs in the EU cosmetics regulations.

Recently, the exploration for the environmental transformation of FMs has drawn increased attention. A few reports have focused on the photochemical behavior of synthetic musks [23], such as musk tibetene [24], musk xylene [25], tonalide [25], and galaxolide [26]. The existing data exhibit that FMs are photochemically unstable, but their complete mineralization seems impossible during environmental transformation, even during advanced oxidation processes [26]. As a result, the generated transformation products may be persistent and impose adverse effects on living organisms. Generally, degradation is often considered as the decrease of toxic process due to the formation of transformation products with more polarity, but recently some transformation products are reported to be more toxic than their parent [27]. For instance, enhanced carcinogenic activity was reported during the environmental transformation of nitro-musks [25]. Increased bioaccumulation and ecotoxicity were observed during the photochemical transformation of polycyclic musk tonalide. Particularly, the generated phenolic product has comparable bioaccumulation with persistent organic pollutants [25]. Besides synthetic musks, some FMs are still carelessly added, including those that have already raised concerns due to their potential environmental and health risks. Significant differences were reported in the mechanisms and toxicity from the photochemical transformation of synthetic musks [28], but they are rarely discussed, even several that are the known cause of skin sensitization adverse effects, such as CA and isoeugenol [29]. Thus, health concerns over these antimicrobial FMs exposure are driven by the research on the environmental transformation and fate of FMs.

The aim of this study was to provide more insights into photochemical transformation mechanisms that could affect the environmental fate and potential toxicity evolution of detrimental antifungal FMs. For this, we performed photochemical transformation of antimicrobial fragrance CA under UV irradiation. The main reactive species were analyzed using laser flash photolysis, and the transformation products were identified by high performance liquid chromatography quadrupole time-of-flight tandem mass spectrometry (HPLC-QTOF-MS). Finally, the aquatic toxicity, irritation, and skin sensitization were assessed to elucidate the photodegradation mechanism and toxicity activity of CA and its byproducts.

## 2. Materials and Methods

### 2.1. Materials

Cinnamaldehyde (CA, 99%) and *p*-Benzoquinone (*p*-BQ, 99%) were purchased from Macklin reagent company. Aceton, methanol and acetonitrile (MeCN) at HPLC grade were obtained from ANPEL Laboratory Technologies (Shanghai, China). Isopropanol (IPA), furfuryl alcohol (FFA), potassium iodide (KI), and triethanolamine (TEOA) were purchased from Aladdin biochemical technology company (Shanghai, China). 5,5-Dimethyl-1-pyrroline N-oxide (DMPO) and 2,2,6,6-Tetramethylpiperidine (TEMP) were bought from the Sigma-Aldrich Trading Co., Ltd. (Shanghai, China). Luminescence of P. phosphoreum were purchased from Institute of Soil Science, Chinese Academy of Sciences (Nanjing, China). Ultrapure water was prepared from a Millipore Milli-Q System (Darmstadt, Germany, resistivity 18.25 MΩ). The purity of Oxygen (O_2_) and Nitrogen (N_2_) was 99.99% for experiment used.

### 2.2. Photochemical and Scavenging Experiments

Steady-state photochemical experiments were conducted in a reactor equipped with a 500 W high-pressure mercury lamp as the irradiation source. A quantity of 30 mL of each sample solution was added into a quartz test tube, which was vertically placed outside the glass well at the fixed distance. The light was filtered through various quartz jackets in order to explore the photochemical properties of CA under different condition, and through the quartz tube containing circulating water which was used for maintaining a constant temperature of 25 ± 1 °C. Simultaneously, the total organic carbon (TOC) was detected using a Shimadzu TOC-5000A TOC analyzer (Kyoto, Japan).

The scavenging experiments were designed to investigate the contribution of the active free radicals by degradation rate of CA. FFA was used to quench singlet oxygen radical (^1^O_2_), and IPA was added to capture the hydroxyl radical (^•^OH). Further, hydrated electron ($e_{\mathrm{aq}}^{-}$) and superoxide anion free radical (O_2_^•−^) were quenched by MeCN and *p*-BQ, respectively. On the other side, the triple excited state was reserved by bubbling N_2_ into the solution, and 10% acetone was used to promote the generation of triple excited states, while TEOA was added to quench the excited state. Approximately 1 mL of each reaction solution was removed at intervals, and the change of CA concentration was further analyzed. The kinetics of the photochemical and scavenging experiments were described with the pseudo-first-order rate constant (k). Each experiment was carried out in triplicate.

### 2.3. Laser Flash Photolysis and Electron Paramagnetic Resonance

Laser flash photolysis (LFP) was used to capture the intermediate species of the reaction of CA solution. LFP was performed using a Nd:YAG solid laser at an excitation wavelength of 266 nm. The average energy of a single pulse was 70 mJ. The 1 cm quartz sample pool was viewed through the analysis light and the laser light in a vertical direction. The monochromator and R955 photomultiplier were used to separate and gather the emergent light, which was then changed into electrical signals. The software processed and presented the signal from the Agilent infinitum model type digital oscilloscope. LFP experiments were performed in the presence of air, N_2_, O_2_, KI, TEOA, and MeCN. All experiments were performed at room temperature, about 25 (±1) °C.

Electron paramagnetic resonance spectra (EPR, Bruker*/emxplus-10/12, Billerica, MA, USA) were used to detect the possible active species in the photodegradation process of CA solutions. Production of active free radical ^•^OH and O_2_^•−^ was monitored using 5,5-Dimethyl-1-pyrroline N-oxide (DMPO) as spin trapping agent. The ^1^O_2_ was detected with 2,2,6,6-Tetramethylpiperidine (TEMP) as spin trapping agent in ethanol solution due to the facile disproportionation in water.

### 2.4. Analytical Methods

High performance liquid chromatography (HPLC: Agilent 1260 series, Santa Clara, CA, USA) was employed to monitor the concentration of CA. Chromatographic separation was achieved on C18 column (5 m, 250 mm × 4.6 mm) with the temperature 25 °C. The mobile phase was 70% methanol in ultrapure water with a flow rate of 0.5 mL·min^−1^, while the injection volume was 20 µL. Detection was achieved through a diode array detector (DAD) at 280 nm.

Before and after irradiation, the photodegradation products were identified through high performance liquid chromatography quadrupole time-of-flight tandem mass spectrometry (HPLC-QTOF/MS, Agilent 1260/6545, Santa Clara, CA, USA). Positive mode electrospray ionization (ESI^+^) was acquired with the initial fragmentor voltage of 175 V. A 10 μL injection volume was analyzed via the Agilent Ecilpse Plus C18 column (column length 2.1 mm, inner diameter 50 mm, particle size 1.78 μm, Santa Clara, CA, USA) under the eluent flow rate of 0.2 mL min^−1^. The mobile phase composition was water (solvent A) and MeCN (solvent B). The following gradient elution program was used: 90:10 at starting time for 3 min; changed linearly to 60:40 over 20 min and keep 5 min; and then back to 90:10 with a conditioning time of 3 min and for 2 min (total time 30 min).

To better determine the structure of transformation products with indeterminate sites, the possible structures were optimized in the Gaussian 09 program package. We employed the 6-311G** basis set for all atoms, combined with the M06-2X functional in aqueous solution (ε = 78.39).

### 2.5. Toxicity Evaluation

To assess the toxicity of the CA solutions, both experimental and theoretical assays were performed. The ecotoxicity was experimentally determined as inhibition of luminescence of P. phosphoreum by reaction solution. As well, the toxicity tests were conducted with Dxy-3 toxicity analyzer (Nanjing, China) [30]. Further, ACD/Percepta platform (Build 2921.10 Jan 2017)—an industry-leading property prediction and lead optimization design tool—was used to predict the irritation to skin and eyes by the compound. Skin sensitization analysis of CA and its byproducts was conducted by Pred-Skin 3.0 software, a tool for the assessment of potential human body skin sensitization with about 89% predictive ability [31].

## 3. Results and Discussion

### 3.1. Kinetic of CA Photochemical Transformation

**Figure 1:**
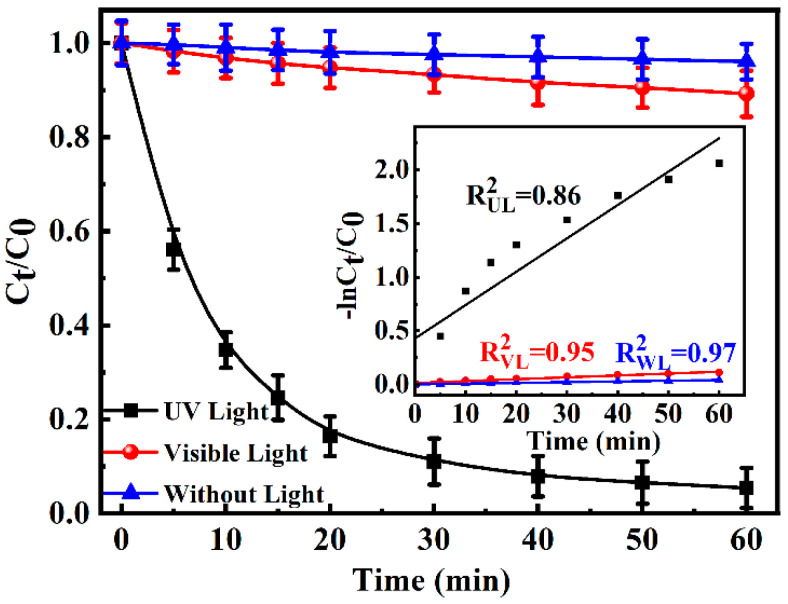
The degradation of 100 μM CA under UV light, visible light, and without light. Inset: The linear degradation of −ln(C_t_/C_0_) versus time for the photo-degradation of CA.

Figure 1 shows the photochemical degradation of 100 μM CA under UV and visible light, as well as without light, respectively. The hydrolysis of CA could be nearly negligible in the absence of light irradiation because of the insignificant decrease (<4%) of CA concentration within 60 min, while the visible light made a weak contribution to the CA photochemical degradation, and 10.8% CA was degraded at the same time. By contrast, 94.6% CA could be quickly eliminated under UV irradiation. As seen in the inset of Figure 1, ln(C_t_/C_0_) exhibited linearity with irradiation time, indicating that the photo-degradation of CA was a pseudo-first-order reaction. The reaction rate constant (*k*) and half-life (t_1/2_) of CA were calculated and listed in Table S1. The degradation rate (*k*) was obtained as 0.059 min^−1^ under UV irradiation, 28 times higher than that under visible irradiation. Meanwhile, the half-life (t1/2) under UV irradiation was obtained as 11.8 min, 0.036 times lower than that under visible irradiation. Therefore, the data implied the photo-degradation of CA could easily occurr under UV irradiation. However, it was worth noting that the mineralization of CA was only 28% when CA was eliminated (Figure S1). Thus, the transformation products and their implication for toxicity should be paid much attention.

### 3.2. Identification of Transformation Products of CA and Toxicity Evaluation

To further understand the environmental fate of CA in water, we identified the transformation products during photo-degradation CA using HPLC-QTOF/MS. By comparison of the spectra with that of control sample without UV irradiation, five unknowns were recorded in solution under UV irradiation. The extracted ion chromatograms (EICs) and the fragment peaks in the second-order mass spectrum (MS/MS) were obtained in Figures S2 and S3, respectively. Additionally, Table 1 summarizes the predicted molecular formulas, retention time, [M + H]^+^, fragment ions, and the corresponding structures.

The protonated molecular mass of 133.064 was detected in 14.4 and 4.0 min under the irradiation of 60 min, considered to be isomer. The retention time of 14.4 min was identified as parent compound CA confirmed with standard substance, while the isomer product in 4 min was considered to be Cis-isomerization (Cis-CA) due to the same fragmentation patterns with CA. The accurate mass of the product 149.059 predicted its molecular formula as C_9_H_8_O_2_ with the retention time of 8.2 min. Analysis of the fragment ions m/z 131.048 indicates the elimination of one water molecule and the addition of an O atom compared with protonated molecular mass of CA. Hence, it was reasonable to tentatively confirm the product as cinnamic acid, which was consistent with the characteristic fragment ions in the literature [32]. With the retention times of 2.9 min and [M + H]^+^ of 105.069, the product molecular formula predicted was C_8_H_8_ and its characteristic fragment ions of m/z 79.054 corresponded to the loss of C_2_H_3_, which was reasonably identified as styrene. The protonated molecular mass of 131.048 eluted at different retention times 6.3 and 9.7 min was detected, which indicated that two products were isomers and its molecular formula was C_9_H_6_O. The similar fragment ions m/z 103.054 indicate the loss of a CHO group. Combined with the rationality of the CA photochemical degradation, the cyclization products were inferred. The product with higher stability was 1-Oxo-1H-indene with the retention time 9.7 min. Conversely, the product with the retention times of 6.3 min was identified 1aH-indeno [1,2-b]oxirene.

**Figure 2:**
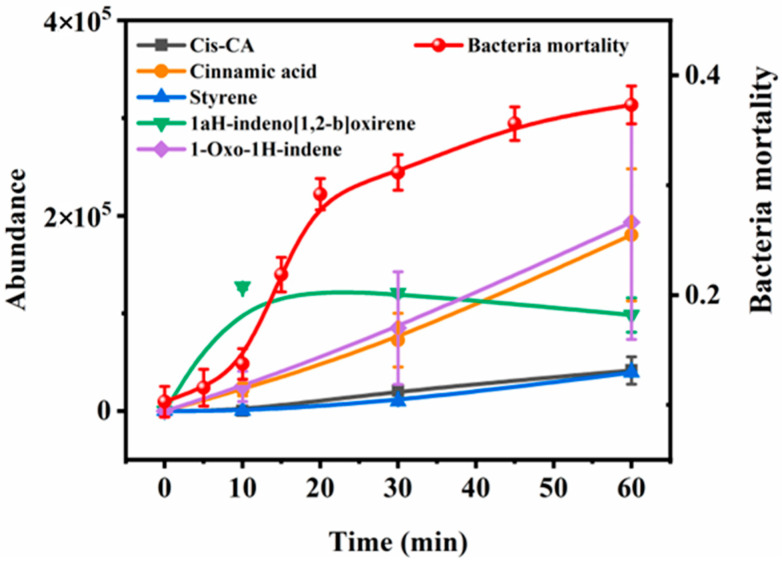
The peak area of the product changes and acute toxicity evaluated with the mortality of photobacterium phosphoreum during the photodegradation of 100 μM CA.

In order to evaluate the potential toxicity of the CA solution on aquatic organisms, we performed the exposure of CA solution on the bright luminescent bacteria and the peak area changes of the product under different photo-degradation time. Figure 2 showed the inhibitory effect of CA solution with different photodegradation time on the bright luminescent bacteria. The inhibition rate of luminescent bacteria increased gradually with the increase of illumination time. After 60 min of illumination, the survival rate of luminescent bacteria decreased from 89.7% to 62.7%, indicating that CA could be transformed into products with more aquatic toxicity during photo-degradation. Correspondingly, the peak areas of the products increased with the prolongation of degradation time, except the product 1aH-indeno [1,2-b]oxirene (Figure 2). Hence, the products of Cis-CA, cinnamic acid, styrene, and 1-Oxo-1H-indene may pose a potential threat to aquatic organisms.

Furthermore, we also evaluated the potential irritation and allergy effects of CA and its products on human health, and the irritation of eyes and skin by the ACD/Percepta platform and Pred-Skin model, respectively. Herein, the probability (*p*) between 0% and 100% was predicted for a tested pollutant to irritate skin and eyes. Seen from Table 2, the *p* values of skin irritation were 69% (Cis-CA), 83% (cinnamic acid), 73% (styrene), 36% (1aH-indeno [1,2-b]oxirene), and 81% (1-Oxo-1H-indene). The products (except 1aH-indeno [1,2-b]oxirene) were more or equally likely to sting skin than the original CA (69%). These data indicate that transformation products could likely impose comparative or more serious skin irritation, particularly the products cinnamic acid and 1-Oxo-1H-indene. What is more, the parent compound CA and its products were also irritating to the eyes, especially the product cinnamic acid (94%) compared to the parent compound CA (75%).

In addition, severe skin sensitization was observed during the photodegradation process to experimental operator (Figure S4). The experiment was conducted by a healthy adult researcher with no prior history of skin conditions. During the photochemical transformation of cinnamaldehyde, minor dermal exposure occurred despite the use of standard protective equipment, including lab coats and gloves. As a result, localized allergic reactions, as shown in Figure S4, were observed. The symptoms initially appeared after 4–5 days of exposure and became more immediate over time, occurring after only 2–3 days. Due to the clear allergic response, no cytokine assessments were conducted out of ethical and safety considerations. The reproducibility and increasing severity of the symptoms strongly suggest a sensitization potential of cinnamaldehyde and its photoproducts, which may interact with the skin’s complex immune system, microbiome, and natural protective barriers. This indicates that cinnamaldehyde and its photoproducts might trigger sensitization responses. These findings support the hypothesis that photochemically generated substances from cinnamaldehyde pose significant skin sensitization risks. Future studies will explore these effects in more detail, using in vitro cell models or animal experiments, and will include cytokine analysis to better understand the underlying mechanisms. These studies will also investigate the influence of the skin’s vitamin D production, UV protection mechanisms (such as melanin), and microbiome in response to these photochemically generated compounds under appropriate ethical approval.

Further, we predicted the sensitizer effects of photolysis products and parent compound CA towards human skin, and the results were listed in Table 2. CA exhibits 99% sensitizer effect on human skin, confirmed by previous experimental research [33,34]. Under UV irradiation, all transformation products could also induce sensitizer effect towards human skin, as indicated by the (+) standing for sensitizer. Furthermore, Figures S5 and S6 display carbon–carbon double bond and carbonyl group that contributed to their sensitizing effect. Therefore, we can conclude that some of the substances produced during the photodegradation of CA are more likely to cause allergies, even more than the parent compounds, making the degraded solution more allergenic. Furthermore, systematic research combining cell experiments or skin tissue models is needed in the future to elucidate the molecular mechanisms underlying the skin health effects induced by transformation products.

### 3.3. Photodegradation Mechanisms

#### 3.3.1. The Identification of Transient Intermediates

**Figure 3:**
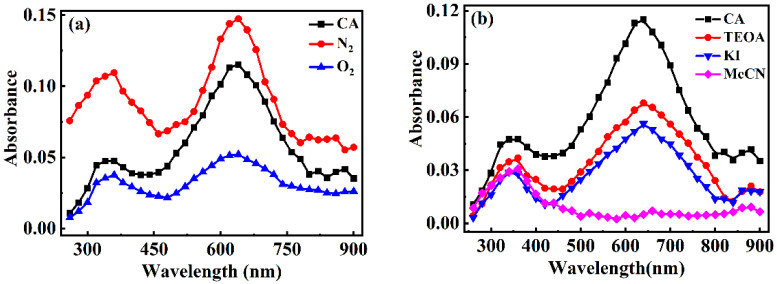
Transient absorption spectra of 100 μM CA in (**a**) N_2_ and O_2_ saturated solution and (**b**) different quenching agents at 32 ns.

In order to obtain more direct evidence for the degradation mechanism, the absorption spectra of transient intermediates were investigated during the photo-degradation of CA using laser flash photolysis. As shown in Figure S7, the transient intermediates were generated and increased with the short lifetime of 30 ns, followed by a prompt attenuation. Two characteristic absorption peaks at 360 nm and 640 nm were observed. According to the spectra from photo-degradation of various structural analogs [35], the maximum absorption at 360 nm could be assigned plausibly as the excited states of CA (^3^CA^*^), and/or the radical cation (CA^•+^). Also, the absorption peak at 640 nm could be hydrated electrons (e_aq_^−^). In order to verify our hypothesis, laser flash photolysis experiments with specific scavengers were designed sophisticatedly. Figure 3 shows the transient absorption spectra in the presence and absence of scavengers. O_2_ can quench the active triplet excited state (e.g., ^3^CA^*^) [36], and react with e_aq_^−^ into O_2_^•−^ [37]. However, the presence of O_2_ can enhance the formation of CA^•+^ acting as an electron acceptor [35]. As shown in Figure 3a, the absorption peak at 360 nm and 640 nm decreased significantly, implying that both ^3^CA^*^ and e_aq_^−^ could be formed in the photodegradation of CA. This conclusion was further confirmed by the observation in solution with the exclusion of O_2_ (Figure 3a).

Additionally, it is noticeable that besides ^3^CA^*^, other transient intermediate with absorption peak at 360 nm must also be formed, because the transient absorbance spectra of ^3^CA^*^ did not completely disappear with adding its quenchers, such as O_2_ (Figure 3a) and TEOA (Figure 3b). In the presence of KI with quenching effect on cationic radicals (CA^•+^), the absorption peaks decreased, confirming that CA^•+^ exists in this photo-degradation of CA. As well, the generation of e_aq_^−^ was also verified in the CA solution containing 20% MeCN (e_aq_^−^ quencher) (Figure 3b), that is, the absorption peak at 640 nm was nearly disappeared. Therefore, ^3^CA^*^, CA^•+^, and e_aq_^−^ were confirmed to be generated in the CA solution under UV irradiation.

#### 3.3.2. The Identification of the Reactive Species

The EPR experiment was used to provide more evidence for the reactive species in this system. As shown in the Figure S8, hydroxyl radical (^•^OH) was detected by EPR in CA solution after irradiation, and the signal increased with the extension of degradation time, indicating that ^•^OH played a role in the photodegradation process of CA. The characteristic signal of O_2_^•−^ was also gradually enhanced, indicating that O_2_^•−^ will be generated in the process of CA photodegradation. At the same time, ^1^O_2_ was captured by the trapping agent TEMP, and its signal was slightly enhanced as shown in Figure S8, indicating that ^1^O_2_ might be generated in this system. Therefore, we can conclude that ^•^OH, O_2_^•−^, and ^1^O_2_ will be generated in the CA solution when irradiated by the UV light.

**Figure 4:**
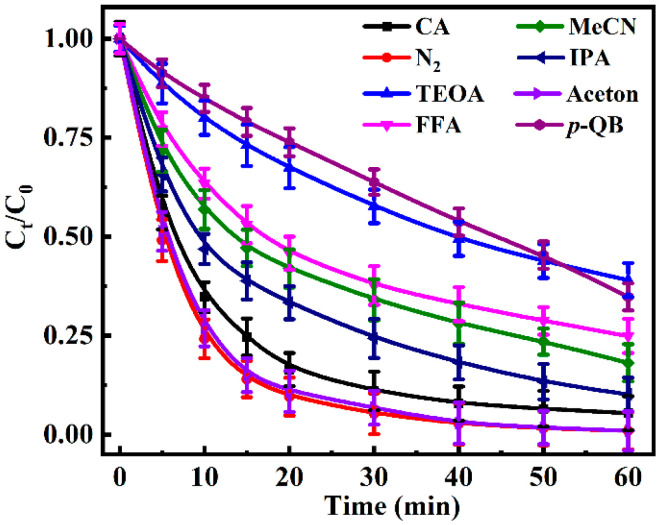
Degradation kinetics of 100 μM CA under UV irradiation (no scavenger, nitrogen saturated, in 10% acetone solution, 100 μM FFA, 5 mM isopropanol, in 20% MeCN solution, 5 mM TEOA and 5 mM *p*-BQ).

A series of quenching experiments were conducted to determine the contribution of different reactive active species in CA photodegradation. Figure 4 showed the degradation kinetics curve, and the degradation rate constants were calculated and summarized in Table S2. When TEOA as ^3^CA^*^ quenching agent [38] was added into CA solution, the photo-degradation rate was significantly inhibited, falling from 0.059 min^−1^ to 0.017 min^−1^. The data suggest that ^3^CA^*^ could play an important role in the photodegradation with contribution rate of 33.6%. On the other hand, in N_2_ saturation and with 10% acetone solution protecting the highly active ^3^CA^*^ [39], the degradation rates increased to 0.086 min^−1^ and 0.084 min^−1^, respectively, further confirming that ^3^CA^*^ participated in the CA photodegradation.

In the presence of *p*-BQ as a quencher of superoxide radical (O_2_^•−^) [40], the degradation rate significantly decreased from 0.059 min^−1^ to 0.016 min^−1^. This result indicates that O_2_^•−^ could participate in the photodegradation reaction of CA with the contribution rate of 29.4%. The addition of FFA (an ^1^O_2_ quencher) [41] also inhibited the photo-degradation of CA, and the contribution rate was calculated as 19.5%. When added to CA solution, MeCN can remove hydrated electron (e_aq_^−^) [42], further slowing down the degradation rate to 0.031 min^−1^. This indicated that e_aq_^−^ would participate the photo-degradation of CA with the contribution rate of 12.7%. As well, the degradation rate of CA after adding isopropanol was 0.042 min^−1^, slightly lower than the rate of CA 0.059 min^−1^, which indicates that ^•^OH may exist in the CA solution but play a minor role (4.7%) in CA photodegradation.

#### 3.3.3. Photochemical Degradation Mechanism of CA

**Figure 5:**
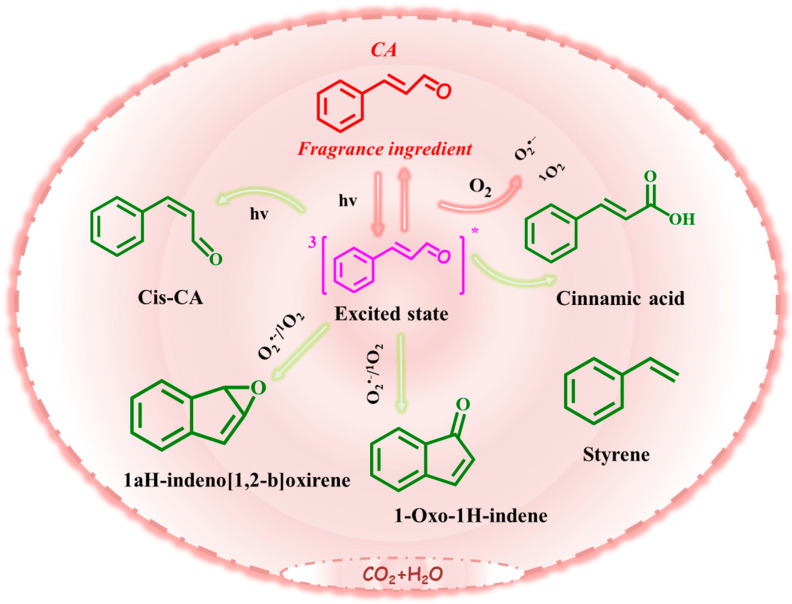
Degradation pathway of CA.

According to the elucidated photodegradation intermediates and products, we had temporarily proposed the pathways of CA photolysis as presented in Figure 5. The photolysis process mainly formed monocyclic aromatic and polycyclic aromatic derivative products, which mainly were controlled by ^3^CA^*^, ^1^O_2_, and O_2_^•−^ in these reactions. CA can absorb the photon energy to form the excited singlet state of CA (^1^CA^*^), then be further transformed to an excited triplet state (^3^CA^*^) due to instability of ^1^CA^*^. The product Cis-CA may be formed through bond rotation of ^3^CA^*^ [43,44]. The^1^CA^*^ and ^3^CA^*^ will be quenched by the ground state molecular oxygen to produce ^1^O_2_. Meanwhile, O_2_^•−^ was easy to find in the presence of O_2_ under illumination. ^3^CA^*^ could be oxidized to form the product cinnamic acid under the presence of ^1^O_2_ or O_2_^•−^ [45]. The product styrene may be formed through decarboxylation from the product cinnamic acid [46]. Simultaneously, the formation of the polycyclic aromatic derivative products 1aH-indeno [1,2-b]oxirene) and 1-Oxo-1H-indene may involve intramolecular cyclization and rearrangement under UV light irradiation. Further, the products were also going to convert to each other in this process. The transformation products of CA were more or less correlated to the probability of eye and skin stimulation. The transformation products all had a sensitizing effect due to the contained carbon–carbon double bond. Hence, the photolysis of CA needs urgent attention, especially the adverse health effects of the transformation products. This study preliminarily explored photochemical risk of antimicrobial fragrances. However, the structural identification, as well as the quantitative analysis of the products and their potential molecular action mechanisms, still needs to be further clarified in future research.

## 4. Conclusions

The photochemical degradation mechanism and toxicity of CA were studied to provide reference for the photodegradation removal of CA in the actual environment. A total of 94.6% CA could be degraded under UV light irradiation after 60 min, and the degradation rate was 0.059 min^−1^. As the quenching experiments, LFP and EPR analysis showed that the main active species in the photodegradation of CA were ^3^CA^*^, O_2_^•−^, and ^1^O_2_, and their contribution rates were 33.6%, 29.4%, and 19.5%, respectively. Meanwhile, e_aq_^−^ and ^•^OH also participated in the reaction. In addition, the degradation products of CA were analyzed by mass spectrometry, which mainly included cis-cinnamyl aldehyde, cinnamic acid, styrene, 1aH-indeno [1,2-b]oxirene), and 1-Oxo-1H-indene. CA can absorb the photon energy to form the unstable excited triplet state (^3^CA^*^), then the product Cis-CA may be formed through bond rotation. The products of styrene, cinnamic acid, 1aH-indeno [1,2-b]oxirene), and 1-Oxo-1H-indene were further formed based on the presence of ^3^CA^*^ and ^1^O_2_/O_2_^•−^. Finally, the acute toxicity experiment on luminescent bacteria showed that the survival rate of the luminescent bacteria decreased from 89.7% to 62.7%, indicating that more toxic substances Cis-CA, cinnamic acid, styrene, and 1-Oxo-1H-indene may be produced after CA photodegradation. Meanwhile, further theoretical verification by ACD/Percepta and Pred-skin showed that the probability of eye and skin stimulation by cinnamic acid was higher than by CA. The transformation products all belonged to sensitized substances, which indicated that CA will still produce sensitized substances after photodegradation, which will have a certain impact on human health.

## Figures and Tables

**Table 1:** Summary the photolysis products of CA by HPLC-QTOF/MS.

Name (Abbreviation)	Formula	Retention Time (min)	[M + H]^+^	Fragment Ions	Structure
Cinnamyl aldehyde (CA)	C_9_H_8_O	14.4	133.064	115.054	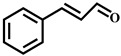
Cis-Cinnamyl aldehyde (Cis-CA)	C_9_H_8_O	4.0	133.064	115.054	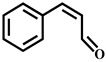
Cinnamic acid	C_9_H_8_O_2_	8.2	149.059	131.048	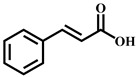
Styrene	C_8_H_8_	2.9	105.069	79.054	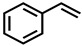
1aH-indeno [1,2-b]oxirene	C_9_H_6_O	6.3	131.048	103.053	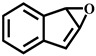
1-Oxo-1H-indene	C_9_H_6_O	9.7	131.048	103.054	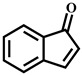

**Table 2:** Stimulus response and skin sensitization analysis of CA and its products by ACD/Percepta and Pred-Skin.

Compounds	Irritation (*p*)		Skin Sensitization (%)	
	Eye Irritation	Skin Irritation	LLNA (In Vivo)	HRIPT/HMT (Human)
CA	75.0	69.0	(+) 99.8	(+) 99.0
Cis-CA	75.0	69.0	(+) 99.8	(+) 99.0
Cinnamic acid	94.0	83.0	(+) 98.2	(+) 86.0
Styrene	68.0	73.0	(+) 99.7	(+) 93.3
1aH-indeno [1,2-b]oxirene	39.0	36.0	(+) 89.6	(+) 97.7
1-Oxo-1H-indene	53.0	81.0	(+) 99.4	(+) 99.2

(+)—sensitizer; LLNA—Local lymph node assay; HRIPT/HMT—Human repeat insult patch test/Human maximization test.

## Data Availability

All data used to generate tables and figures are available in the main document or the Supplementary Materials of this paper, and available on request to the corresponding author.

## Supplementary Materials

The following supporting information can be downloaded at https://www.mdpi.com/article/10.3390/toxics13050386/s1. Figure S1: The evolution of TOC removal efficiencies during the photodegradation of 100 μM CA; Figure S2: The extracted ion chromatograms (EICs) of products; Figure S3: The product fragment peaks in the second-order mass spectrum; Figure S4: Skin irritation caused by exposure to cinnamaldehyde during the experiment; Figure S5: Skin sensitization probability map of human repeat insult patch test/human maximization test (HRIPT/HMT) in vivo by pred-skin; Figure S6: Skin sensitization probability map of local lymph node assay (LLNA) in vivo by pred-skin; Figure S7: The transient absorption spectra at different time intervals of 100 μM CA solution; Figure S8: The analysis of the active species (^•^OH, O_2_^•−1^, ^1^O_2_) in the CA solution by EPR; Table S1: The degradation efficiency, pseudo-first-order rate constant *k*_1_, and half-life for CA with different light conditions; Table S2: Experimental condition, purpose, pseudo-first-order rate constant *k*_1_, and half-life for CA with different experimental conditions.
