# Supplementary material for: Overlooked Photochemical Risk of Antimicrobial Fragrances: Formation of Potent Allergens and Their Mechanistic Pathways
Source: Toxics. 2025 May 10;13(5):386. doi: 10.3390/toxics13050386 (PMC12115848; doi:10.3390/toxics13050386)
Supplement: Supplementary file 1 [file toxics-13-00386-s001.zip › toxics-3603704-supplementary.pdf]

Supplementary Material:

Overlooked Photochemical Risk of Antimicrobial Fragrances: Formation of Potent Allergens and Their Mechanistic Pathways

Xiaolin Niu[1,2,#], Junji Wu[1,3,#], Yi Chen[1,2], Na Luo[1,2], Yanpeng Gao[1,2,*]

[1]*Guangdong-Hong Kong-Macao Joint Laboratory for Contaminants Exposure and Health, Guangdong Key Laboratory of Environmental Catalysis and Health Risk Control, Institute of Environmental Health and Pollution Control, Guangdong University of Technology, Guangzhou 510006, China;*

[2]*Guangzhou Key Laboratory Environmental Catalysis and Pollution Control, Guangdong Basic Research Center of Excellence for Ecological Security and Green Development, School of Environmental Science and Engineering, Guangdong University of Technology, Guangzhou 510006, China;*

[3]*Shantou Polytechnic, Department of Natural Sciences, Shantou, 515078, China*

*Corresponding Author: **Prof. Yanpeng Gao** E-mail: gaoyp2016@gdut.edu.cn

Table S1. The degradation efficiency, pseudo-first-order rate constant k_1 and half-life for CA with different light conditions.

Experiment condition	Degradation efficiency (%)	k_1 (min^{-1})	Half-life (min)
UV light	94.6	0.059	11.8
Visible light	10.8	0.0021	330.1
Without light	3.9	0.0007	990.2

Table S2. Experimental condition, purpose, pseudo-first-order rate constant k_1 and half-life for CA with different experimental conditions

Experiment condition	Experiment objective	k_1 (min^{-1})	Half-life (min)	Degradation rate (%)	Contribution rate (%)
CA	For contrast	0.059	11.8	94.6	--
N_2	Keep the triple excited state	0.086	8.0	99.0	--
10%Aceton	Triple excited photosensitize	0.084	8.2	99.0	--
TEOA	Quenching the excited state	0.017	40.1	61.0	33.6
p-BQ	Quenching $O_2^{\bullet-}$	0.016	42.5	65.2	29.4
FFA	Quenching $^1O_2$	0.027	25.9	75.1	19.5
MeCN	Capture e_{aq}^-	0.031	22.1	81.8	12.7
IPA	Quenching ·OH	0.042	16.4	89.9	4.7

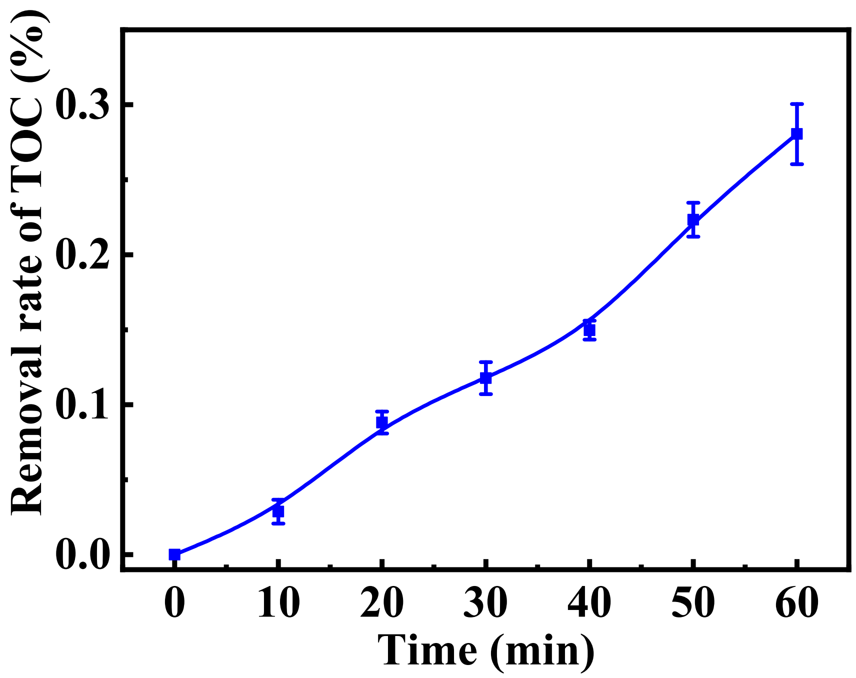

Figure S1. The evolution of TOC removal efficiencies (%) during the photodegradation of 100 μM CA.

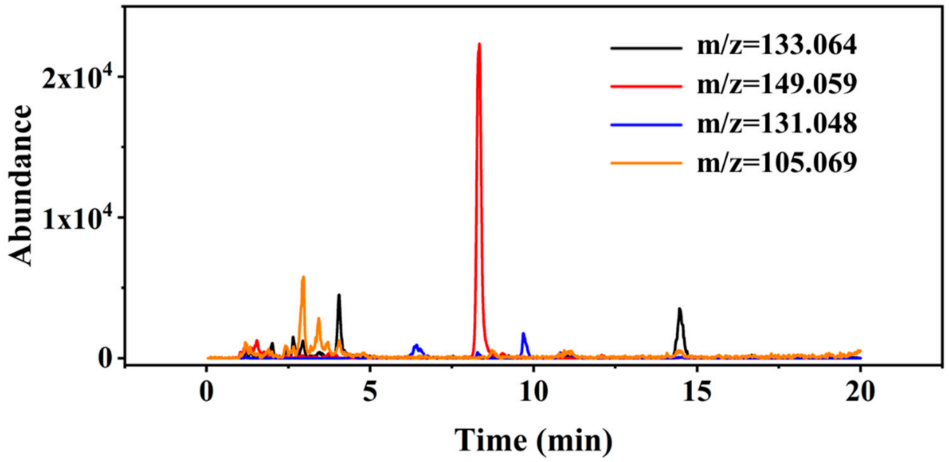

Figure S2. The extracted ion chromatograms (EICs) of products.

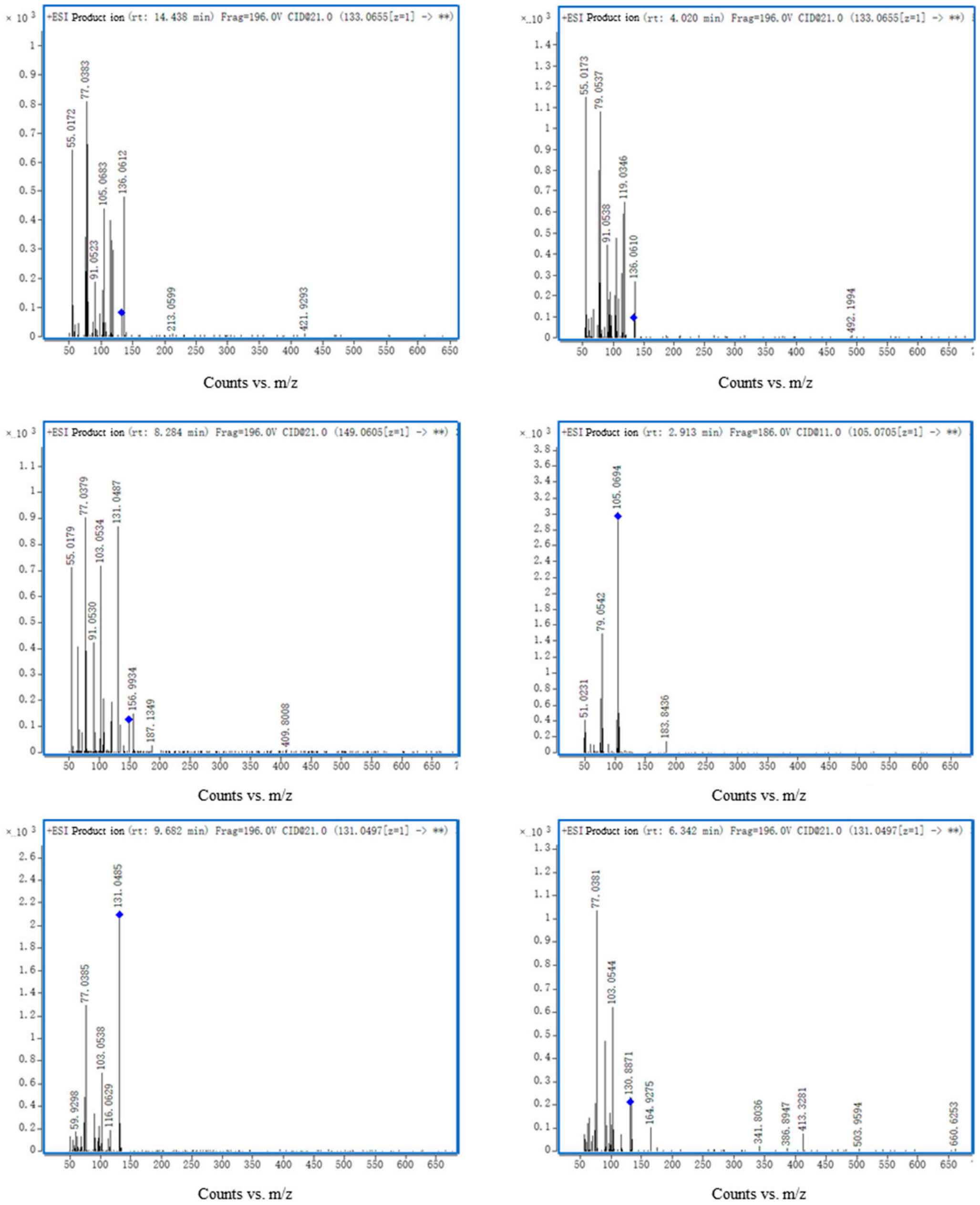

Figure S3. The product fragment peaks in the second - order mass spectrum

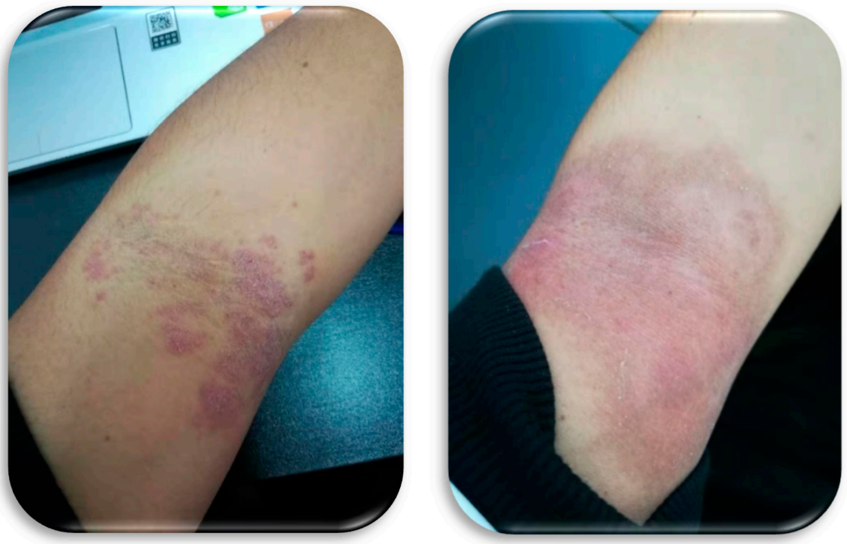

Figure S4. Skin irritation caused by exposure to cinnamaldehyde during the experiment.

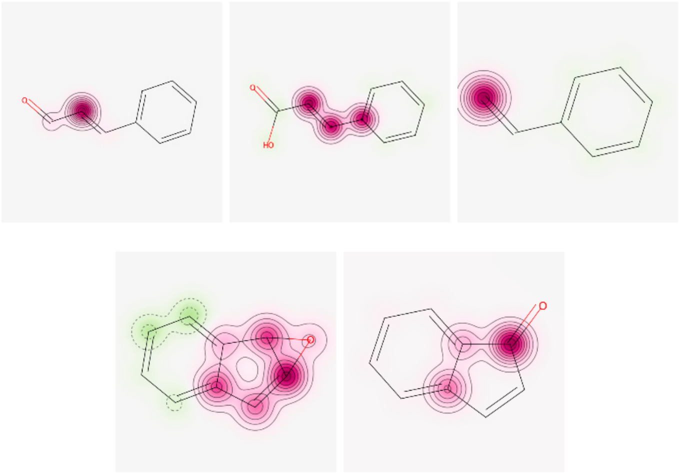

Figure S5. Skin sensitization probability map of human repeat insult patch test/human maximization test (HRIPT/HMT) in vivo by pred-skin.

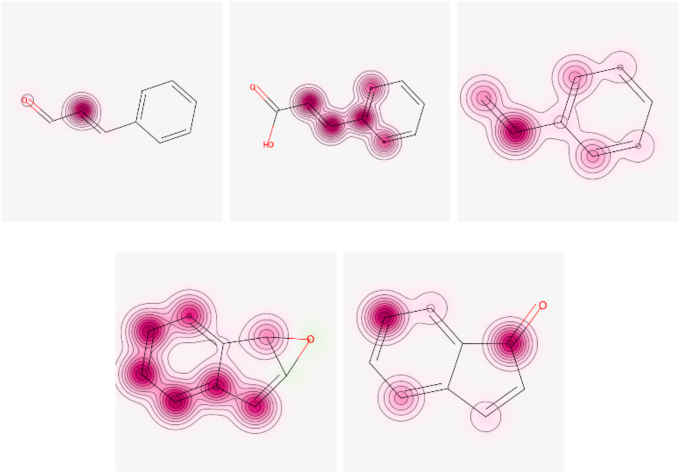

Figure S6. Skin sensitization probability map of local lymph node assay (LLNA) in vivo by pred-skin.

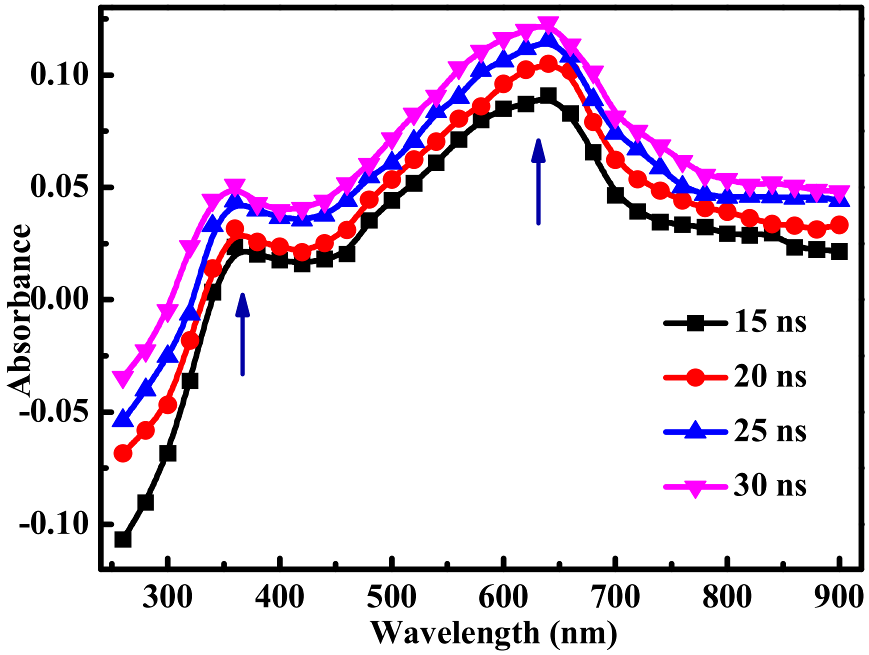

Figure S7. The transient absorption spectra at different time intervals of 100 μM CA solution.

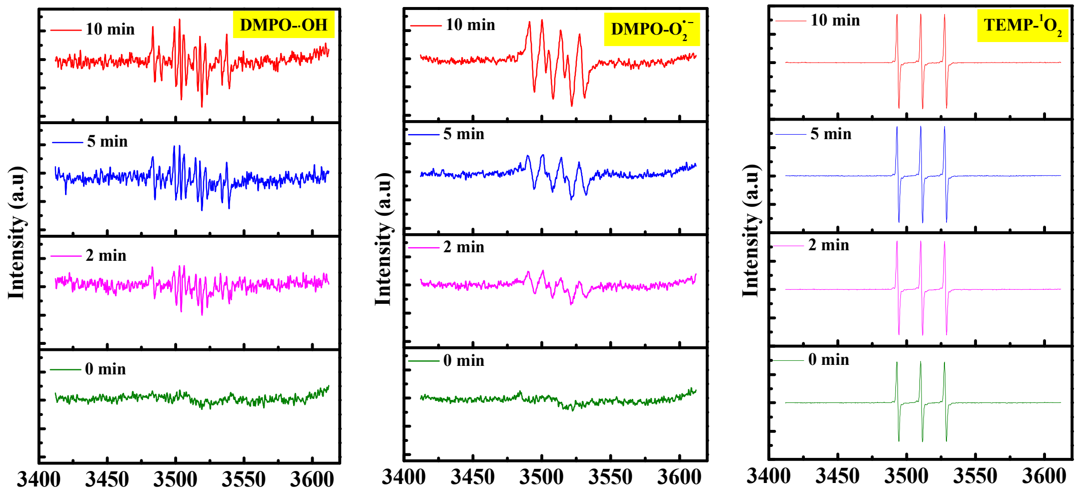

Figure S8. The analysis of the active species ($^{\bullet}OH$, $O_2^{\bullet-}$, $^1O_2$) in the CA solution by EPR.
